# Comparison of rectal swab, glove tip, and participant-collected stool techniques for gut microbiome sampling

**DOI:** 10.1186/s12866-020-02080-3

**Published:** 2021-01-14

**Authors:** Meghan I. Short, Robert Hudson, Benjamin D. Besasie, Kelly R. Reveles, Dimpy P. Shah, Susannah Nicholson, Teresa L. Johnson-Pais, Korri Weldon, Zhao Lai, Robin J. Leach, Bernard Fongang, Michael A. Liss

**Affiliations:** 1 University of Texas Health San Antonio, Glenn Biggs Institute for Alzheimer’s and Neurodegenerative Diseases, San Antonio, TX USA; 2 Department of Urology, University of Texas Health San Antonio, 7703 Floyd Curl Drive, San Antonio, TX 78229 USA; 3 The University of Texas at Austin, College of Pharmacy, Austin, TX USA; 4 Department of Population Health Sciences, University of Texas Health San Antonio, San Antonio, TX USA; 5 Department of Surgery, University of Texas Health San Antonio, San Antonio, TX USA; 6 University of Texas Health San Antonio, Genome Sequencing Facility, Greehey Children’s Cancer Research Institute (GCCRI), San Antonio, TX USA; 7 Department of Cell Systems and Anatomy, University of Texas Health San Antonio, San Antonio, TX USA; 8 Department of Biochemistry and Structural Biology, University of Texas Health San Antonio, San Antonio, TX USA

**Keywords:** Gut microbiome, Stool collection, Rectal swabs, Digital rectal examination, 16 s rRNA gene sequencing

## Abstract

**Background:**

Studies of the gut microbiome are becoming increasingly important. Such studies require stool collections that can be processed or frozen in a timely manner so as not to alter the microbial content. Due to the logistical difficulties of home-based stool collection, there has been a challenge in selecting the appropriate sample collection technique and comparing results from different microbiome studies. Thus, we compared stool collection and two alternative clinic-based fecal microbiome collection techniques, including a newer glove-based collection method.

**Results:**

We prospectively enrolled 22 adult men from our prostate cancer screening cohort SABOR (San Antonio Biomarkers of Risk for prostate cancer) in San Antonio, TX, from 8/2018 to 4/2019. A rectal swab and glove tip sample were collected from each participant during a one-time visit to our clinics. A single stool sample was collected at the participant’s home. DNA was isolated from the fecal material and 16 s rRNA sequencing of the V1-V2 and V3-V4 regions was performed. We found the gut microbiome to be similar in richness and evenness, noting no differences in alpha diversity among the collection methods. The stool collection method, which remains the gold-standard method for the gut microbiome, proved to have different community composition compared to swab and glove tip techniques (*p*< 0.001) as measured by Bray-Curtis and unifrac distances. There were no significant differences in between the swab and glove tip samples with regard to beta diversity (*p*> 0.05). Despite differences between home-based stool and office-based fecal collection methods, we noted that the distance metrics for the three methods cluster by participant indicating within-person similarities. Additionally, no taxa differed among the methods in a Linear Discriminant Analysis Effect Size (LEfSe) analysis comparing all-against-all sampling methods.

**Conclusion:**

The glove tip method provides similar gut microbiome results as rectal swab and stool microbiome collection techniques. The addition of a new office-based collection technique could help easy and practical implementation of gut microbiome research studies and clinical practice.

**Supplementary Information:**

The online version contains supplementary material available at 10.1186/s12866-020-02080-3.

## Background

The intestinal microbiome may play a role in the pathogenesis of many types of cancer and other diseases, thus providing a modifiable biomarker with potential for treatment interventions, which can complement standard screening programs [1–4]. The current gold standard for fecal microbiome collection is to obtain participant-collected stool with a home-based collection kit [5–7]. Unfortunately, participants may not adhere to the directions or send in the sample. For example, only 60% of participants returned their at-home collected stool specimens in a cohort at high risk for colorectal cancer [8]. Stool sample collection can be challenging due to participants’ comfort level, inconsistent sample collection, and increased collection cost [5, 6, 9–12]. Capturing samples during a clinic visit would save time and potentially increase the number of participants in a study, while reducing bias due to systematic differences between participants who do and do not send in samples or follow the directions for proper collection.

Studies have found that microbiome sample collection via rectal swabs versus participant-collected stool identify similar microbial composition [9, 10, 12]. We have recently published a study comparing exam gloves after a digital rectal exam (DRE) to swab methods for fecal material collection for microbiome studies [13]. We noted similar DNA yield and quality using glove tips compared to rectal swab techniques in different cohorts, yet did not perform both tests on the same participant. Herein, we used the 16S rRNA gene sequencing method to test the collection of fecal specimens with the office-based post-DRE glove tip and rectal swab, and participant-collected stool techniques, using the same participants to perform a paired investigation regarding the similarities and differences in microbial communities between these techniques.

## Results

### Population

We enrolled 22 individuals from whom we obtained three gut microbiome samples collected by different methods (participant-collected stool, rectal swab, post-DRE glove tip) from 8/2018 to 4/2019. All participants were men previously enrolled in a longitudinal observational prostate cancer screening study, with a median age of 74.5 years. We display the demographics for the cohort in Table 1.

**Table 1:** Demographics Table

Variable	N (%)
**Race**	
White	20 (90.9)
Black	2 (9.1)
**Ethnicity**	
Hispanic or Latino	7 (31.8)
Not Hispanic or Latino	15 (68.2)
**Age**	
58–69	5 (22.7)
70–79	14 (63.6)
80–89	3 (13.6)
Median	74.5
**BMI**	
< 25.0	5 (22.7)
25.0–35.0	12 (54.5)
> 35.0	5 (22.7)
Median	30.26
**Smoking History**	
Never smoked	14 (63.6)
Former smoker	8 (36.4)
**Total**	22

### Alpha and Beta diversity

**Fig. 1:**
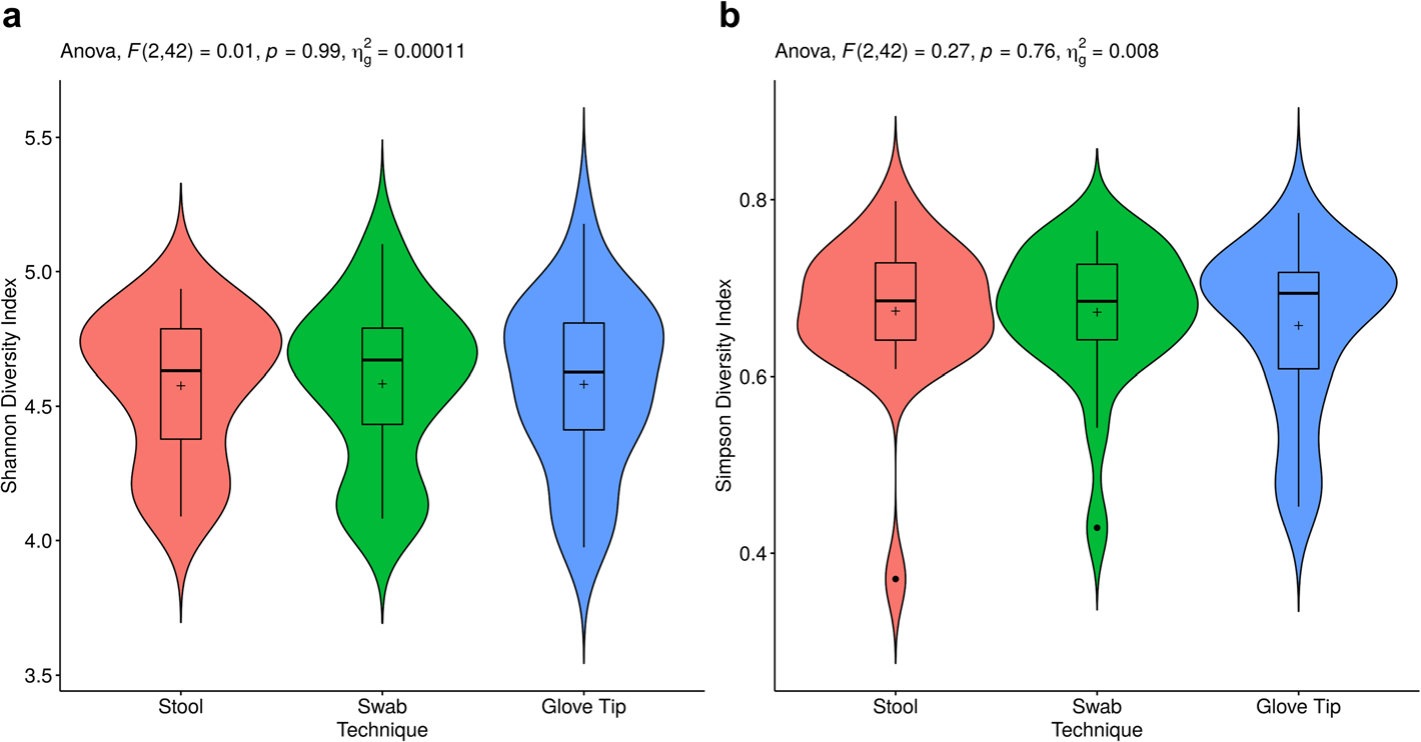
Simpson and Shannon alpha diversity index assessment. Box denotes the first and third quartiles, whiskers denote the most extreme observations within 1.5 times the inter-quartile range distance from the box; outliers are marked as points. The horizontal bar within the box represents the median, and the “+” denotes the mean. The red, green, and blue areas represent kernel probability densities for the data. Repeated measures ANOVA found no significant differences in Shannon (*p*=0.99) or Simpson (*p*=0.76) diversity indices among the sample collection methods

**Fig. 2:**
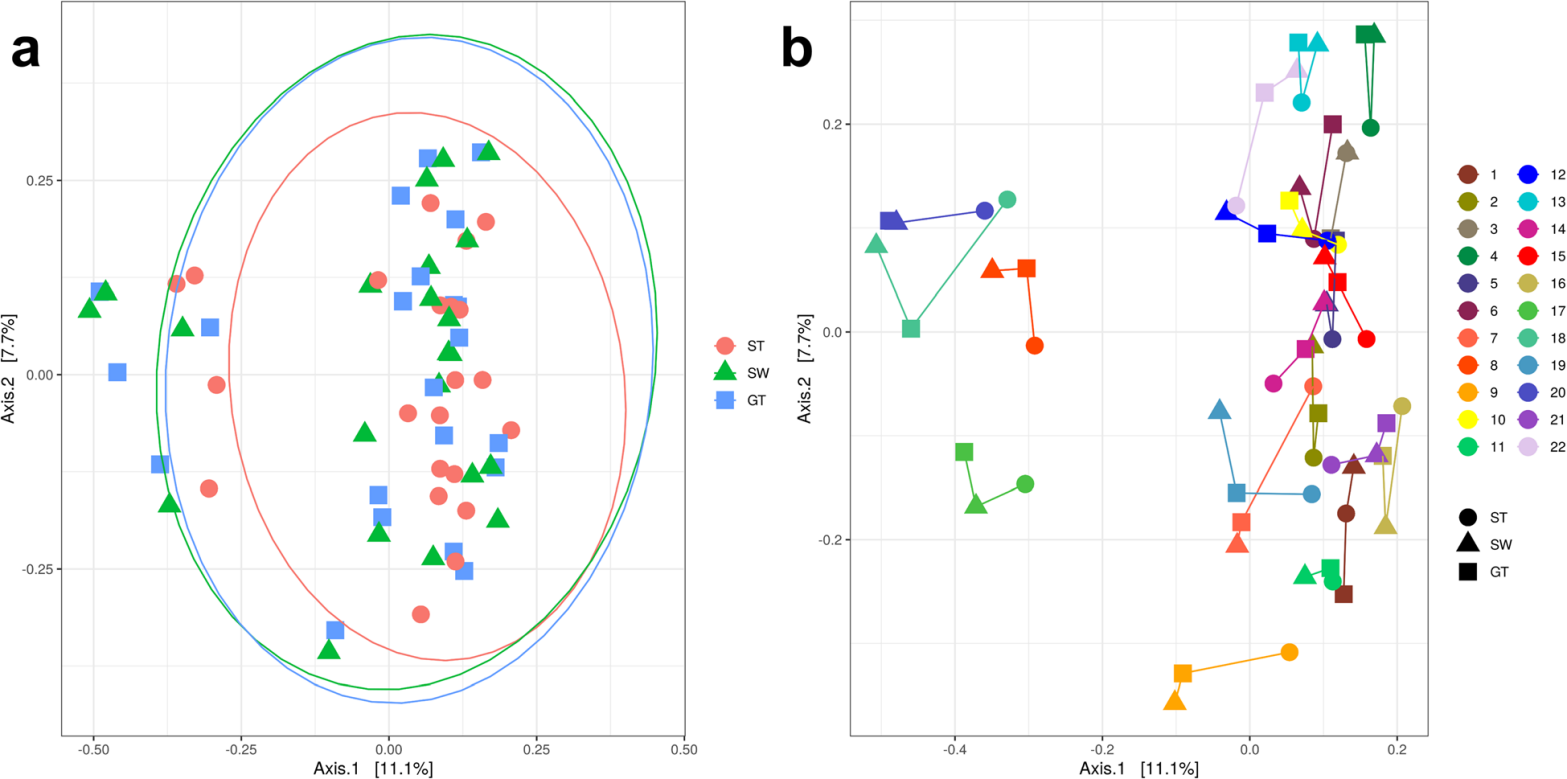
Principal Coordinates Analysis (PCoA) using Bray-Curtis distances. (**a**) Principal coordinates analysis (PCoA) plot based on Bray-Curtis distances by sampling method with data ellipses based on multivariate t distributions (**b**) Principal coordinates analysis (PCoA) plot based on Bray-Curtis distances by person and method

At the operational taxonomic unit (OTU) level, we found no difference in the Shannon (mean [SD] for participant-collected stool: 4.58 [0.27], swab: 4.58 [0.30], glove tip: 4.58 [0.31], *p*=0.99) or Simpson (mean [SD] for participant-collected stool: 0.67 [0.08], swab: 0.67 [0.08], glove tip: 0.66 [0.09], *p*=0.76) index of alpha diversity among the collection methods (Fig. 1) [14]. Regarding the beta diversity, there were differences among the collection methods in Bray-Curtis distances (*p*< 0.001; Fig. 2). In particular, in pairwise comparisons with *p*-values adjusted using the Benjamini-Hochberg approach, stool samples differed from swab (*p*< 0.001) and glove tip (*p*< 0.001); swab and glove tip samples did not differ significantly from one another (*p*=0.59). We also identified this pattern in tests of unifrac distances, both unweighted (global *p*< 0.001, swab vs. stool p< 0.001, glove tip vs. stool p< 0.001, glove tip vs. swab *p*=0.69) and weighted (global *p*< 0.001, swab vs. stool p< 0.001, glove tip vs. stool p< 0.001, glove tip vs. swab *p*=0.26). Secondary analyses examining the V1-V2 and V3-V4 regions separately had similar results. For both the V1-V2 region and the V3-V4 region, there were significant differences among the methods globally (*p*< 0.001 in each region), again driven by differences of stool compared with swab (p< 0.001 in each region) and glove tip (p< 0.001 in each region) methods, with no differences between the swab and glove tip methods (*p*=0.36 for V1-V2 region, *p*=0.20 for V3-V4 region). We present a PCoA plot with Bray-Curtis distances by technique (Fig. 2a) and by sampling technique and participant (Fig. 2b) for the mixed V1-V2 and V3-V4 region data, where there is proximity among samples from the same person, despite statistically significant differences among the methods.

**Fig. 3:**
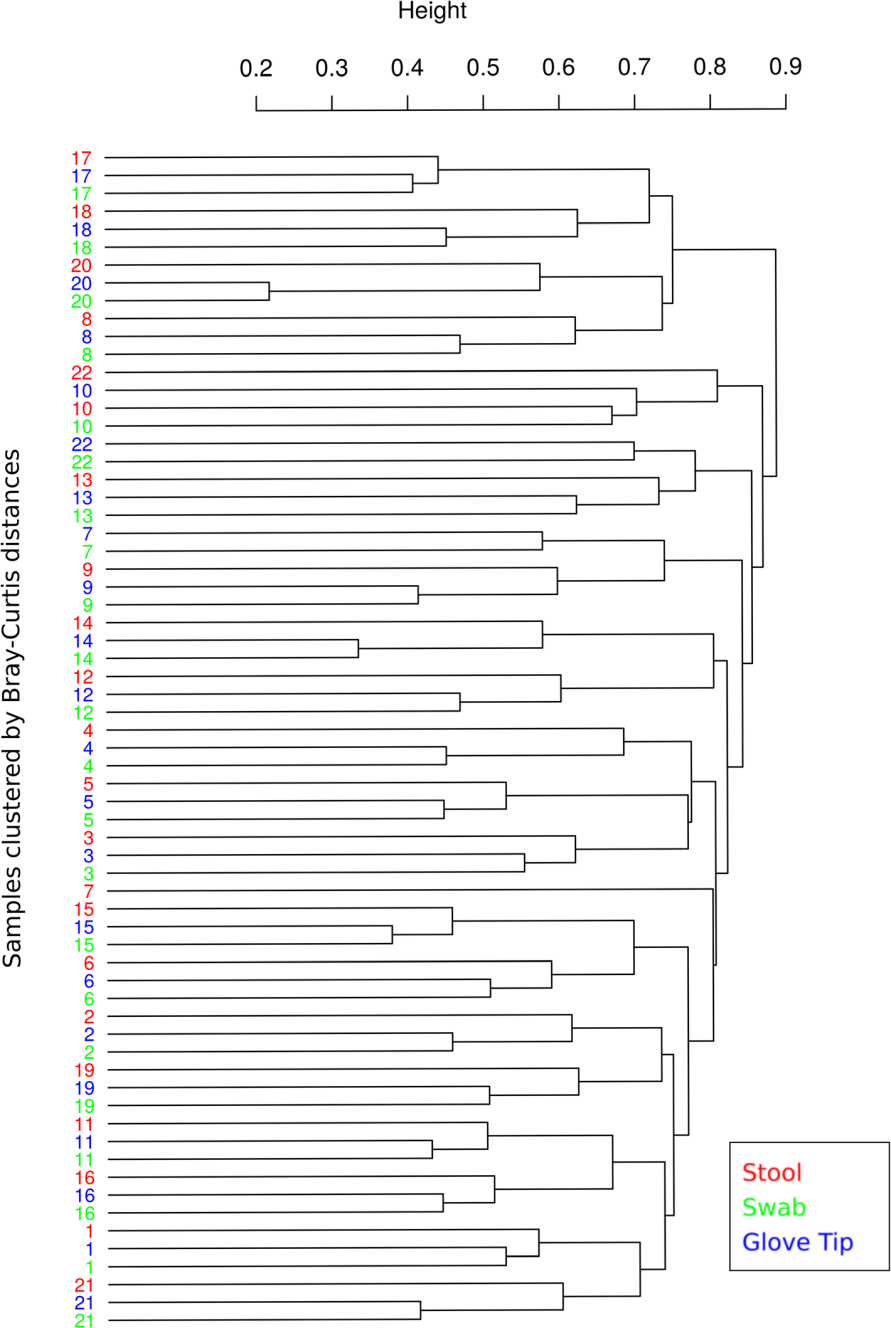
Dendrogram of samples clustered based on Bray-Curtis distances. Bray-Curtis distances were calculated at the OTU level and clustered using the unweighted pair group method with arithmetic mean (UPGMA) algorithm. The numbers represent the individual patients and the sample is represented by color (Red = Stool, Green = Swab, and Blue = Glove tip)

Most samples clustered together based on the person sampled from a hierarchical clustering procedure based on Bray-Curtis distances, as shown in the dendrogram (Fig. 3). If two participants (9% of the overall sample) were excluded (IDs 7, 22), the remaining 20 would each have their three measurements cluster together within a clade. Overall, 21 out of 22 people (95%) showed closer grouping between swab and glove tip samples relative to stool collection; the remaining person had stool and swab measurements more closely related.

### Bacterial abundance analysis

**Fig. 4:**
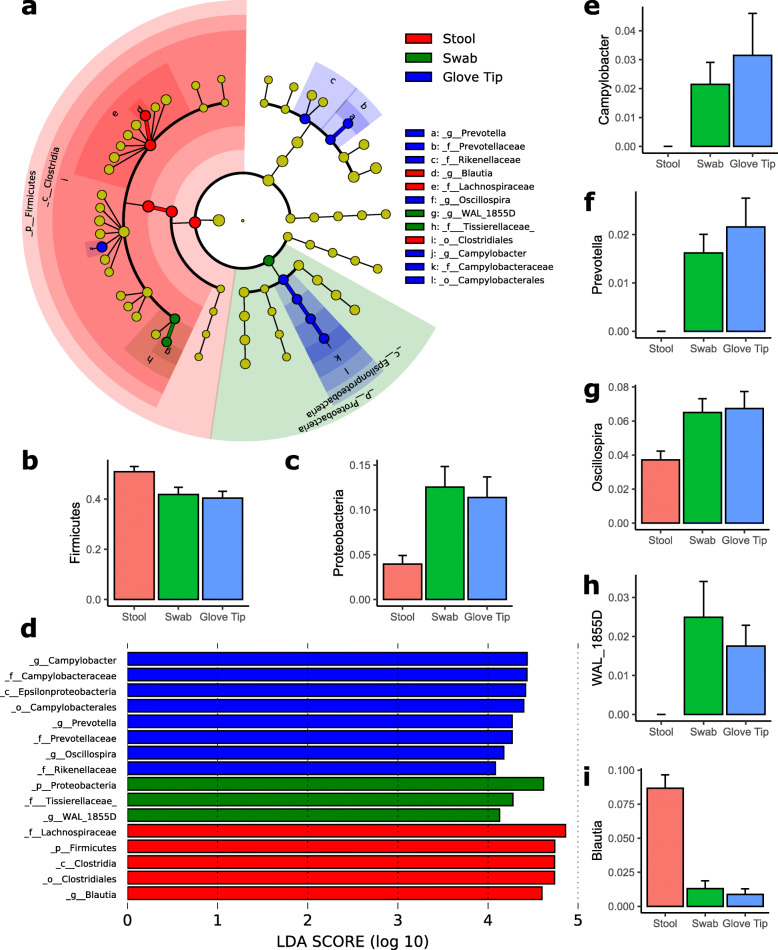
Summary of taxa which were differentially expressed. (**a**) Cladogram of taxa which were differentially expressed based on Linear discriminant analysis Effect Size (LEfSe), comparing each collection method against the other two combined (“one against all”). There were no significantly different taxa identified when testing “all against all” sampling methods. (**b**)-(**c**) Relative abundance of Firmicutes and Proteobacteria by sampling method. (**d**) Linear discriminant analysis (LDA) scores indicating the effect size of each differentially expressed taxon. (**e**)-(**i**): Relative abundances of differentially abundant genera by sampling method

Analysis of differentially abundant taxa using Linear Discriminant Effect Size (LEfSe) analysis [15] found no significantly differently abundant taxa using the all-against-all algorithm, which identifies differences among all pairwise comparisons and therefore employs a more stringent multiple comparisons adjustment. We performed a secondary analysis with a lower effect size threshold of 2.0, and there were still no significantly differently abundant taxa using the all-against-all algorithm. Using the one-against-all algorithm, which compares abundance in each category to abundance in the other categories collectively and therefore requires a less stringent multiple testing correction, we identified the major phylum components unique to each technique. We found an enrichment of Firmicutes in the stool sample collection and Proteobacteria in the swab collection technique (Fig. 4). Within Firmicutes, the genus *Blautia* was enriched in stool samples, the genus *Oscillospira* was enriched in glove tip samples, and the genus *WAL 1855D*, a Sporobacterium, was enriched in swab samples. A cladogram, a plot of linear discriminant analysis (LDA) scores, and relative abundances of phylum and genus signatures of each method are displayed in Fig. 4. Absolute abundances of genera by phylum and sampling method are presented in Supplementary Fig. 1.

## Discussion

Our prospective, observational study, in which we obtained gut microbiome samples from healthy men using three techniques, showed an overall similarity between collection methods, especially at the participant level. Alpha diversity measured by Shannon’s and Simpson’s index did not significantly differ among the techniques examined. Beta diversity differed between stool samples and each of the other two collection methods; we did not observe differences between swab and glove tip collection techniques. LEfSe analysis showed that some taxa were differentially abundant among sampling methods when using a less stringent one-against-all testing scheme, but not with a more stringent all-against-all testing scheme. While examining different regions can lead to differences in abundance and taxa identified [16–18], we found similar results when examining the V1-V2 and V3-V4 regions separately in secondary analyses of beta diversity. Studies employing swab or glove tip collection methods should be aware that community composition may differ between these methods and stool sample collection.

Our study is consistent with previous literature noting similarities in microbiome analysis between the stool and rectal swabs [9, 19]. A hospital-based study of eight participants measured one stool and several swab samples taken over the course of a day, and found that between-person variation in beta diversity significantly exceeded within-person variation [9]. Our study found similar results, evidenced by the clustering of samples within participants based on Bray-Curtis distances. Another study by Jones et al. [20] compared stool with swab samples and mucosa biopsies, finding some taxa to vary between stool and swab samples, including Campylobacteracea and Prevotellaceae, which we observed to be over-represented in glove tip samples (which correlated highly with swab samples) and which the previous study found over-represented in swab samples. The study additionally found other families whose prevalence varied between stool and swab samples, which our study did not identify, and one family (Rikenellaceae) which had an opposite direction of effect. However, the Jones et al. study sample comprised people with a history of colon polyps, and overall gut microbial community structures may differ between their participants and ours. The Jones et al. study, and others, suggest that rectal swabs may represent more mucosal taxa than stool samples do, which may explain some differences between the sampling methods [21–23]. Mucosal taxa are not generally captured in stool, and can only be fully sampled using invasive mucosal biopsies. Given the similarities in our current study between glove tip and swab samples, it may be that glove tip samples capture mucosal species, possibly avoiding the need for mucosal biopsies in some cases. Our work is the first comparative study to include the glove tip technique, and we do not compare directly with mucosal biopsies, so further studies are needed to test this hypothesis.

The glove tip gut microbiome collection technique performed at the time of a clinical rectal exam is simple, requires no participant preparation, and samples can be transported easily from the clinic to the laboratory. The glove tip technique attempts to improve the implementation of microbiome studies and microbiome-based testing as compared to the more cumbersome at-home stool collection, which is complicated to collect and is participant-dependent. In some clinical settings, providers perform a standard-of-care digital rectal exam as part of cancer screening; use of the glove tip acquisition method means no other acquisition procedures, such as a rectal swab, are needed to obtain a sample for gut microbiome assessment. While stool samples remain the “gold standard” of gut microbiome assessment, our results suggest that inter-individual differences are still adequately captured using the glove tip technique.

There are limitations to our study. The sample size was relatively modest, leaving the possibility that there were differences in microbial communities among the collection methods that we did not detect. Specifically, stool collections were home based and swab and glove tips were collected in clinic, with differences being the swabs and glove tips were transferred to the lab within 4 h, but stool was placed immediately into the omigene kits to preserve DNA. All subjects were healthy, so we were unable to assess whether microbiome biomarkers for prostate cancer risk are as useful when measured using the glove tip method compared to other methods. However, similarities in diversity and lack of differentially expressed taxa under strict multiple testing criteria suggest that the methods’ results will be similar overall. Another limitation is that the material obtained using the glove collection method is small and may not be able to be used for metabolomic analysis; however, it provided enough sample for 16 s rRNA evaluation. Glove tips and swab samples were obtained in the same clinical visit, whereas the stool samples were collected at home, such that some differences may be attributed to timing of collection. Finally, providers may perform rectal exams less frequently in women’s health, and so the glove tip method may not be as useful in women’s health. Providers do use fecal occult testing for colorectal screening in both sexes; however, we did not test this particular technique.

## Conclusions

We compare a new glove-based microbiome sample collection method to existing rectal swab and participant-collected stool sample collection methods. Concerning microbial diversity and taxonomic abundance, the glove tip collection is similar to the swab collection technique and generally similar to home-based stool collection. This new collection method, which can be conducted during clinic visits, has the potential to reduce barriers to gut microbiome collection and help implement microbiome sampling in clinical research and practice.

## Methods

### Study population

We attained approval by the Institutional Review Board at the University of Texas Health San Antonio (HSC20000030H). After approval, we prospectively enrolled men from our San Antonio Biomarkers of Risk (SABOR) prostate cancer screening cohort. In this sub-study, we collected rectal swabs, exam glove tips used during digital rectal exam (DRE), and participant-collected stool samples for comparison during a one-time collection period.

### Sample collection

#### DRE glove collection

We used a double-glove technique where the urology provider wore two non-sterile latex gloves during DRE as described in Besasie et al. [13]. Briefly, we cut the glove tip from the outer glove and placed it in phosphate-buffered saline (PBS) in a 5 mL conical tube. We then stored the glove specimen at 4 °C and transported it to the laboratory. The glove tip was then placed in a 2 mL microfuge tube; the PBS solution from the collection tube was transferred to the 2 mL tube and used to rinse the glove tip to remove all collected fecal material. We then removed the glove tip to store the fecal material in the PBS at − 20 °C until DNA isolation.

#### Rectal swab collection

The provided used a swab at the time of DRE with an individual packet of sterile lubricating jelly. The provider placed the rectal swab in a 15 mL sterile centrifuge tube containing 1 mL of PBS. Personnel stored the swab specimen at 4 °C during transport to the laboratory. After delivery to the lab, the fecal swab was removed from the collection tube and the PBS solution was transferred into a 2 mL microfuge tube. The cotton portion of the swab was scraped into the microfuge tube using a sterilized scalpel blade. We stored the swab and PBS material at − 20 °C until DNA isolation.

#### Stool collection

We provided participants with the OMNIgene®-GUT stool collection kit (DNA Genotek, Ottawa, Canada) and instructions for home collection of stools. Stool collections occurred within median of 3 days of the clinic visit with 6 subjects within 24 h and two subjects at the latest of 5 days. The OMNIgene kits allow for DNA preservation at room temperature. The stool was either shipped to the lab or the patient brought the sample to one of our research coordinators. Once in the lab, the specimens were processed identically.

### DNA isolation and quantification

For sample input, we attempted to provide a visible amount of stool on the glove tips and swabs. DNA was isolated from fecal samples using our standard operating procedure (see Appendix of Besasie et al. [13]). We performed a purification of genomic DNA from these respective fecal samples using the QIAamp® Fast DNA Stool Mini Kit according to the kit protocol (Qiagen, Germantown, MD). The DNA concentration was measured using the Thermo Scientific NanoDrop.

### 16S rRNA sequencing

Genomic DNA was used for amplification of V1-V2 variable region of the 16S rRNA genes with custom-designed primers (F27/R534, Youssef 2009, Applied and Environmental Microbiology), and V3-V4 variable region of the 16S rRNA genes following the Illumina 16S metagenomic library preparation guide. Final libraries were quantified, normalized, pooled together, and sequenced by Paired-end sequencing (2 × 300 bp) using Illumina Miseq platform. The average of 264,727 raw pair reads per sample were generated with read length of 300 bp. The sequences were exported as FASTQ files.

### Statistical analysis

Sequence processing was performed using QIIME2 software, and statistical analysis was done using R (version 3.6). Dada2 was used to trim and join paired-end sequences, denoise, dereplicate, and remove chimeras from the sequence data. Forward and reverse reads were truncated to preserve Phred quality scores of 28 in 75% of reads at each base. Taxonomic units (OTUs) were assigned using the *classify-sklearn* feature classifier, which was trained against the Greengenes database with a 97% identity threshold. We removed mitochondria from our sample, and we removed OTUs with fewer than four reads in less than 10% of samples, and samples were rarefied to 2409, the minimum library size after filtering. We compared the groups using the Shannon index and Simpson’s index measures of alpha diversity at the OTU level by conducting a repeated-measures analysis of variance (ANOVA) accounting for differences between individuals [14]. If a global test result was significant, we used paired t-tests to make pairwise comparisons and adjusted *p*-values for multiple testing using the Benjamini-Hochberg procedure. We performed a beta diversity assessment with principal coordinates analysis using Bray-Curtis and weighted and unweighted unifrac distances at the OTU level. We then used permutational analysis of variance (PERMANOVA) implemented in the vegan package to test for differences in community composition among the collection techniques, stratified by each participant [24]. If the global test result was significant, we conducted pairwise tests between groups, and p-values were adjusted using the Benjamini-Hochberg procedure. We used Linear discriminant analysis Effect Size (LEfSe) to identify taxa, which were differentially abundant among the collection technique groups, using LDA score threshold of 4.0 and an alpha level of 0.05, at the genus level and above [25]. Both the all-against-one and all-against-all algorithms within the LEfSe package were employed. Our team used the Huttenhower Lab implementation of LEfSe on the Galaxy platform [15].

The UPGMA clustering algorithm was performed based on Bray-Curtis distances at the OTU level among the samples and was used to plot a dendrogram of the samples.

## Supplementary Information

**Additional file 1.** Bar plot of genera within phyla by sampling method

## Data Availability

The datasets used and/or analyzed during the current study are available from the corresponding author on reasonable request.

## Abbreviations

SABOR: San Antonio Biomarkers of Risk for prostate cancer
DRE: Digital Rectal Exam
PCoA: Principal Coordinates Analysis
DNA: Deoxyribonucleic Acid
rRNA: Ribosomal Ribonucleic Acid
QIIME: Quantitative Insights Into Microbial Ecology
ANOVA: Analysis of Variance
OTU: Operational Taxonomic Unit
PERMANOVA: Permutational Analysis of Variance
LEfSe: Linear Discriminant analysis Effect Size
