# Supplementary material for: Comparison of rectal swab, glove tip, and participant-collected stool techniques for gut microbiome sampling
Source: BMC Microbiol. 2021 Jan 14;21:26. doi: 10.1186/s12866-020-02080-3 (PMC7809826; doi:10.1186/s12866-020-02080-3)
Supplement: Supplementary file 1 — Additional file 1. Bar plot of genera within phyla by sampling method [file 12866_2020_2080_MOESM1_ESM.docx]

**Supplementary Figure 1:** Bar plot of genera within phyla by sampling method


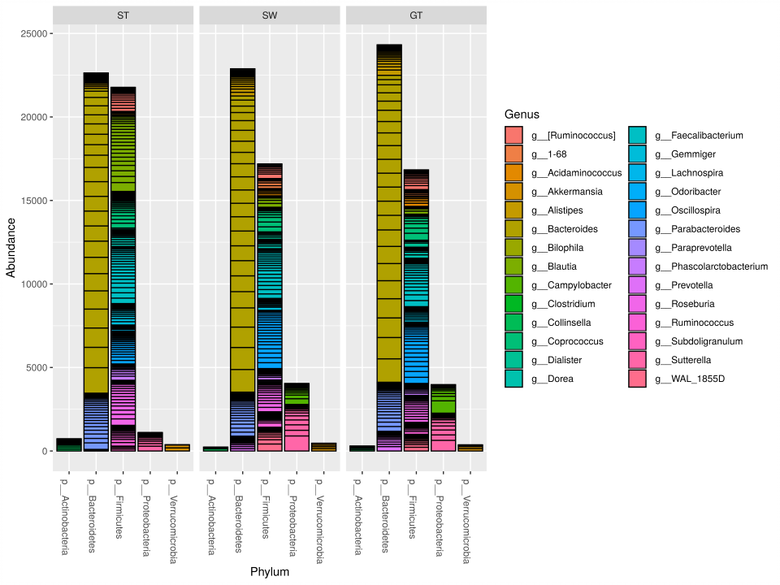


Each black-outlined rectangle represents the abundance of the corresponding genus in one sample; there are 22 blocks (one for each participant) for each genus in each panel chart. ST: stool, SW: Swab, GT: Glove Tip
